# The metabolic demands of internal medicine residency

**DOI:** 10.1186/s12995-019-0234-0

**Published:** 2019-05-06

**Authors:** Sarah Sy, Karanvir Sall, Erika Dempsey, Gale Tedder, Kenneth Michael Madden

**Affiliations:** 1 Department of Geriatric Medicine, 2775 Laurel Street, 7th Floor, Vancouver, BC V6H 0A5 Canada; 2 Department of Geriatric Medicine, Bridgeland Seniors Health Clinic, 1070 McDougall Road NE, Calgary, Alberta T2E 8B8 Canada

**Keywords:** Metabolic parameters of residency, Work hours, Sleep efficiency, Internal medicine

## Abstract

**Background:**

North American and European accreditation bodies have legislated progressively more strict work hour restrictions for residents in light of evidence that sleep deprivation leads to increased medical errors and decreased wellbeing. The purpose of the study is to determine the physiologic demands of internal medicine training during residency as well as document average sleep (on- and off-call) and physical activity performed using accelerometers.

**Methods:**

A total of 40 internal medicine residents working on the clinical teaching unit at a single center were enrolled in the study from November 2011 to March 2016. There were 22 subjects that completed the study and were included in the analysis. SenseWear PRO 2 armband monitors were worn for 5 consecutive days including one call day. The primary outcomes of the study were to quantify and compare the calories per day, steps per day, METs per hour, hours of activity, hours of sleep, and sleep efficiency for on call versus post-call and non-call days.

**Results:**

The average activity per day, calories per day, steps per day and METs per hour for the call day were 7.6 ± 7.6 h, 2647.0 ± 541.1, 11,261.1 ± 2355.9, and 1.7 ± 0.2 respectively. Each of these parameters had a statistically significant F statistic compared to post-call and non-call days. The subjects had a mean of 1.8 ± 2.0 h of sleep per day with a sleep efficiency of 77.3 ± 23.8% for the call day. The F statistic for sleep per day was significant with a *p* value < 0.001.

**Conclusion:**

This study shows that overnight call has a substantial impact on multiple metabolic parameters. These findings have potentially important implications on future resident working hour restrictions.

## Background

Over the past decade, there has been increasing pressure on health systems to decrease resident work hours. The original push to reform resident working hours derived from the case of Libby Zion, an 18-year-old woman who passed away under the care of exhausted residents [1]. There has been increasing evidence that sleep deprivation leads to increased medical errors and threatens resident safety [2–5].

North American and European accreditation bodies have legislated progressively more strict work hour restrictions. In the United States, the Accreditation Council for Graduate Medical Education (ACGME) implemented on July 1, 2017 changes to resident duty-hours based on recent studies that showed non-inferiority of flexible, less-restrictive duty hours compared to standard duty hour policy [6, 7]. The ACGME has capped the work week to 80 h per week averaged over 4 weeks. In Europe, work hour restrictions tend to be even more conservative, with 48 h per week being the latest maximum [3]. In 2011, a Quebec labour arbitrator found that 24-h call shifts violate the Canadian Charter of Rights and Freedoms and limited shift length for all Quebec residents to 16 hours [8]. It is uncertain how this will affect work hour restrictions across Canada but data regarding the physical exertion, caloric expenditure and amount of sleep experienced on call will certainly be helpful in the formulation of national, provincial or institutional policies in this regard.

During their general internal medicine rotation, residents at Vancouver General Hospital (VGH) work in clinical teaching units (CTU) that are comprised of a senior resident (second or third year internal medicine residents), two first year residents (one from internal medicine and one from another specialty), and two to three medical students. The residents have graduated responsibility and participate in a 26 h on-call system. In total, there are 5 CTU teams, and one member from each team is on call each night such that there is a senior resident, 2 junior residents, and 2 to 3 medical students. The purpose of this study is to determine the physiologic demands of internal medicine training during residency as well as document average sleep (on- and off-call) and physical activity performed using accelerometers.

## Methods

### Study population

A study was designed to determine the physiological demands of general medicine training at a single quaternary care centre (Vancouver General Hospital) from November 2011 to March 2016. Ethics approval was obtained through the University of British Columbia Ethics Board. The participants were post-graduate years (PGY) one to three residents on the inpatient internal medicine clinical teaching service, who were recruited by email invitations and posters. The investigators were members of the internal medicine program but at no point were on service at the same time or had any educational authority over the residents.

To be eligible for inclusion into the study, the resident must be working 5 consecutive days on the clinical teaching unit including one call shift, and had not previously participated in this study. The residents were excluded if they did not work 5 consecutive days, or if they were taking modafinil.

### Study protocol

The participants met individually with an investigator to obtain written consent and receive instructions. Anthropomorphic information was self-reported and included sex, age, height, weight, and race. The subjects were instructed to wear SenseWear PRO 2 armband monitors for 5 consecutive days including one call day.

On non-call days, daytime responsibilities for the residents were from 7:45 AM to 5 PM and varied depending on their role as senior or junior resident. The junior residents cared for 6 to 8 patients on the team and participated in educational teaching sessions with the senior resident and/or attending physician. The senior resident was responsible for overseeing the care of the CTU team (20 patients), and teaching the junior residents and medical students. After 5 PM, the residents were dismissed from clinical duties. The on call day consisted of being first call to the ward during the daytime from 7:45 AM to 6 PM followed by doing internal medicine consults from the emergency department from 6 PM to 7:45 AM. Subsequently, the post-call day started at 7:45 AM whereby the residents would finish reviewing new patients and other patient issues with an attending physician until 10 AM, at which point, they were excused from clinical duties and allowed to go home. The residents would return to work the following day.

The SenseWear PRO 2 armband is a non-invasive, wearable monitor that employs multiple sensors including a biaxial accelerometer, heat flux, temperature, and galvanic skin response sensors with artificial intelligence capabilities. The armband is programed with the subject’s sex, age, height, and weight using BodyMedia SenseWear Professional 8.1 Software (Philadelphia, USA). When worn, the armband sensors measure in 1 min intervals and use BodyMedia’s algorithms to determine daily energy expenditure, step count, hours of activity, and METs as well as information regarding sleep patterns [9]. The SenseWear Pro 2 armband recorded physical activity in 1-min epochs when METs were equal to or greater than 1.0. It is worn over the right triceps muscle and participants in previous validation studies have not found it bothersome or to interfere with free-living activity [10, 11]. The armband was permitted to be removed during contact with water, but not more than 60 mins at a time. After completion of the 5 days, the participants met with an investigator to return the device and obtain a summary of their SenseWear data.

### Statistical analysis

Data analysis was conducted after all participants had completed the study. The work days were measured in 24 h periods (e.g. from 7:45 AM to 7:44 AM the next day). The primary outcomes of the study were to quantify and compare the calories per day, steps per day, average METs per hour, hours of activity, hours of sleep, and sleep efficiency for on call versus post-call and non-call days. The SenseWear device algorithm calculated values for each data variable in one minute intervals. These one-minute intervals were tallied over the defined 24 h period (7:45 AM to 7:44 AM) to obtain a value for each day. Next for each variable, an average was created for non-call days, which would allow for comparison to the call day. The data of the subjects that completed the study was analyzed using R version 3.4.2 (R Foundation for Statistical Computing, Vienna, Austria) ANOVA one-way repeated measures to determine if there was a statistically significant difference when on call versus not on call.

## Results

**Fig. 1:**
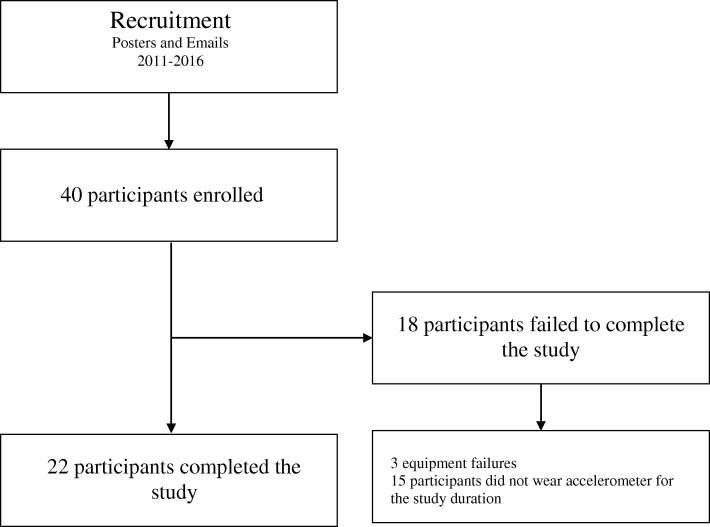
Recruitment Flow Chart

A total of 40 subjects were enrolled in the study from November 2011 to March 2016. The prolonged enrolment period was in part due to the absence of a study investigator to recruit new participants over a two year period. The periods of active recruitment primarily occurred over 10 months from a pool of 150 potential resident participants. There were 22 subjects that completed the study and were included in the analysis (Fig. 1). The mean age was 31.3 years and the majority of the subjects were internal medicine residents. The average number of hours worked in a 5 day period was 52.2 h. Table 1 summarizes the subject demographics. The reasons for not completing the 5 day research study included: intolerance to the device (uncomfortable, bulky, and/or ruined clothing), and battery failure.

**Table 1:** Subject Demographics

	*n* = 22
Age	31.4 ± 6.3
BMI	23.2 ± 4.4
Female (%)	64% (*n* = 14)
Hours worked during 5 day period	52.3 ± 6.3
Ethnicity (%)	
Caucasian	46% (*n* = 10)
Asian	36% (*n* = 8)
Other	18% (*n* = 4)
Specialty (n)	
Internal Medicine	20
Off-service	2
Residents per year (%)	
Junior (PGY-1)	59% (*n* = 13)
Senior (PGY-2/3)	41% (*n* = 9)
Smoker (%)	0%

Metabolic parameters including activity per day, calories per day, steps per day, METs per hour, hours of sleep per day, and sleep efficiency were measured using the SenseWear PRO 2 armband monitors. In terms of activity per day, the call day had a mean of 7.6 ± 7.6 h and the ANOVA analysis showed a statistically significant *p* value < 0.001 compared to the post call and non-call days (Table 2). The calories per day, steps per day and average METs per hour for the call day were 2647.0 ± 541.1, 11,261.1 ± 2355.9, and 1.7 ± 0.2 respectively. Each of these parameters had a statistically significant F statistic compared to post-call and non-call days.

**Table 2:** Metabolic Parameters and One-Way Repeated Measures ANOVA

	Activity (hours per day)	Calories (per day)	Steps (per day)	METS (per hour)	Sleep (hours per day)	Sleep efficiency (%)
Call Day	7.6 ± 7.6	2647.0 ± 541.1	11,261.1 ± 2355.9	1.7 ± 0.2	1.8 ± 2.0	77.3 ± 23.8
Post Call Day	5.0 ± 5.3	2265.8 ± 485.3	8086.8 ± 2952.3	1.4 ± 0.1	8.7 ± 2.1	82.6 ± 6.6
Non Call Day	5.9 ± 5.8	2440.7 ± 531.4	10,099.2 ± 4749.2	1.5 ± 0.2	5.9 ± 1.2	81.2 ± 7.5
F-statistic	13.469	21.491	7.387	8.674	86.127	1.068
*P* value	< 0.001*	< 0.001*	0.002*	< 0.001*	< 0.001*	0.356

With respect to sleep, on the call day, subjects had a mean of 1.8 ± 2.0 h of sleep per day with a sleep efficiency of 77.3 ± 23.8%. The F statistic for sleep per day was significant with a *p* value < 0.001.

## Discussion

This study demonstrates that overnight call has a substantial impact on multiple metabolic parameters. The post-call and non-call days demonstrate decreased physical activity, METs per hour, steps per day, and calories per day compared to the call day. In addition, sleep is significantly reduced on the call day to the point where most residents were achieving only 1–2 h of total sleep in 24 h. Subsequently, the residents required more sleep post call.

While there is a plethora of literature that focuses on resident duty hours, there is a paucity of literature examining specifically the effect of call on metabolic parameters in physicians and residents. Our study is in congruence with previous studies examining the effect of call on sleep. A study by Amirian et al. examining surgeons using actigraphy found call day to be associated with reduced sleep [12]. In addition, this study showed that on post-call day one there was the lowest amount of daytime activity and the highest amount of reduced wake time. Another study by Rose et al. with internal medicine residents on a 1-in-4 call scheduled found reduced sleep on call, but no significant increases in sleep on post call day one [13].

With respect to sleep efficiency, our study reveals decreased sleep efficiency during the call day versus the non-call days. There are few studies that specifically investigate the effect of call on sleep efficiency. A study by Morhardt et al investigated sleep efficiency in urology residents during home call using Fitbit technology [14]. The study found that the residents had 47% sleep efficiency and that the Stanford Sleepiness Scale did not correlate well with the results. In a different study by Ko et al., sleep patterns of urology residents participating in different call schedules were investigated [15]. This study found that there was no difference between day shift, night shift, and 24 h call for total sleep time, light sleep, deep sleep or number of times woken. However, sleep latency was the longest in those residents participating in the day shift call. Thus far, our study is the first to report decreased sleep efficiency during call in internal medicine residents. There is a clinical trial underway examining the impact of night float on sleep patterns in anesthesiology residents (NCT03325244).

Other studies focusing on parameters such as calories per day, METs per hour, and hours of activity could not be found.

In regard to creating policies for duty hour regulation, governing bodies have not taken into account the physiological effects of working extended shifts or overnight call. The literature used to determine policies have focused primarily on measures of well-being, cognitive function, sleep efficiency, and fatigue. Our study revealed that internal medicine residents experienced less hours of sleep and decreased sleep efficiency while on call. Previous studies have shown that lack of sleep contributes to increase errors and worse well-being [4, 5, 7]. In our study, the residents were less physically active on their post-call and non-call days. They did not achieve the recommended amount of exercise per week nor intensity of exercise [16]. Research has shown that sedentary lifestyles can increase the risk of cardiovascular disease, metabolic syndrome, and all cause mortality [17]. Even short bouts of sedentary activity have been shown to have risks [18]. Therefore, the effect of working hours on physiologic and metabolic parameters may require more attention.

Our study has several potential limitations. Slow recruitment resulted in data collection over a wider period of time, which can introduce unaccounted variables. In addition, the small sample size, single site study, short study period, and limitation to primarily internal medicine residents make the generalizability of results to residents from different specialties difficult. Another limitation of the study is that the residents’ baseline physical activity, the number of consults completed during call and the requirement for frequent patient reassessments based on illness severity could not be controlled. As a result, this could impact the parameters measured and may be a reason for the large standard deviations. We were also unable to control which day the residents decided to take call during the study period due to service requirements and personal preferences. Furthermore, sleep quality scores, cognitive performance measures, subjective measures of well-being, and direct observation were not recorded.

## Conclusions

In this single center study of internal medicine residents, we show that overnight call has a negative impact on metabolic parameters including activity per day, calories per day, steps per day, METs per hour, hours of sleep per day, and sleep efficiency. Further research of resident duty hours that incorporates metabolic parameters, patient outcomes measures, and qualitative measures would be beneficial to better understand its impact on residents and implementing sustainable policy changes.

## Abbreviations

ACGME: Accreditation Council for Graduate Medical Education
CTU: Clinical teaching unit
PGY: Post-graduate year
